# Transcatheter aortic valve replacement-in-transcatheter aortic valve replacement for high-risk anatomies: demonstrating the feasibility of index leaflet overhang in a first-in-human case report

**DOI:** 10.1093/ehjcr/ytae529

**Published:** 2024-09-24

**Authors:** Tommaso De Ferrari, Marco B Ancona, Vittorio Romano, Luca Ferri, Filippo Russo, Eustachio Agricola, Antonio Esposito, Matteo Montorfano

**Affiliations:** Interventional Cardiology Unit, IRCCS San Raffaele Scientific Institute, Via Olgettina, 60, 20132 Milan, Italy; Interventional Cardiology Unit, IRCCS San Raffaele Scientific Institute, Via Olgettina, 60, 20132 Milan, Italy; Interventional Cardiology Unit, IRCCS San Raffaele Scientific Institute, Via Olgettina, 60, 20132 Milan, Italy; Interventional Cardiology Unit, IRCCS San Raffaele Scientific Institute, Via Olgettina, 60, 20132 Milan, Italy; Interventional Cardiology Unit, IRCCS San Raffaele Scientific Institute, Via Olgettina, 60, 20132 Milan, Italy; Cardiovascular Imaging Unit, IRCCS San Raffaele Scientific Institute, Via Olgettina, 60, 20132 Milan, Italy; Vita-Salute San Raffaele University, Via Olgettina, 58, 20132 Milan, Italy; Vita-Salute San Raffaele University, Via Olgettina, 58, 20132 Milan, Italy; Clinical and Experimental Radiology Unit, Experimental Imaging Center, IRCCS San Raffaele Scientific Institute, Via Olgettina, 60, 20132 Milan, Italy; Interventional Cardiology Unit, IRCCS San Raffaele Scientific Institute, Via Olgettina, 60, 20132 Milan, Italy; Vita-Salute San Raffaele University, Via Olgettina, 58, 20132 Milan, Italy

**Keywords:** Case report, TAVR in TAVR, Leaflet overhang, Coronary obstruction, Coronary access

## Abstract

**Background:**

Transcatheter aortic valve replacement (TAVR)-in-TAVR within index supra-annular transcatheter heart valves (THVs) and high-risk anatomy can compromise coronary perfusion and re-access.

**Case summary:**

An 80-year-old male presented with acute heart failure caused by degeneration of an Evolut R THV, leading to severe aortic regurgitation. Aortic computed tomography imaging revealed a high-risk anatomy characterized by the index THV’s commissural plane being placed above the coronary ostia and a valve-to-aorta distance of less than 2 mm, resulting not eligible for redo-TAVR according to recent literature. The current case represents the first-in-human application of redo-TAVR with low SAPIEN 3 THV implantation within an Evolut R THV obtaining index leaflet overhang, preserving coronary perfusion and potential coronary re-engagement.

**Discussion:**

Following *in vitro* study, this case demonstrates *in vivo* feasibility of redo-TAVR with low SAPIEN 3 THV implantation within an Evolut R THV obtaining index leaflet overhang.

**Learning points**

- To recognize the anatomical details at the CT scan that enable a safe and effective redo-TAVR
- To select the most appropriate THV and implantation height based on patient-specific anatomy and pathophysiology
- To demonstrate the feasibility and effectiveness of redo-TAVR with low SAPIEN 3 THV implantation within an Evolut R THV obtaining index leaflet overhang

## Introduction

Two decades after the first transcatheter aortic valve replacement (TAVR), we are now facing prosthesis dysfunction requiring reinterventions. Preprocedural planning with computed tomography (CT) scan is mandatory for TAVR-in-TAVR and aims to minimize the risk of acute coronary artery occlusion and preserve future coronary access. Key elements of planning involve assessing the level of coronary ostia, index transcatheter heart valve (THV) expansion, valve-to-aorta (VTA) distances, and calculating the neoskirt height.^1–3^ The neoskirt is created jailing the index THV leaflets between the two THV frames, and its upper edge establishes the critical level below which blood and coronary catheter cannot cross after a redo-TAVR. Depending on the frame height and position of the second THV, index THV leaflets might not be entirely captured between the two THVs, resulting in leaflet overhang, affecting coronary artery perfusion, access, and THV hydrodynamic performance.^4,5^ The highest risk of coronary obstruction and impaired coronary access exists when the index THV commissural plane is located above the coronary ostia, and the VTA distances measure less than 2 mm, aligning with a standard 6 Fr percutaneous coronary intervention (PCI) catheter size.

## Case summary

**Figure 1:**
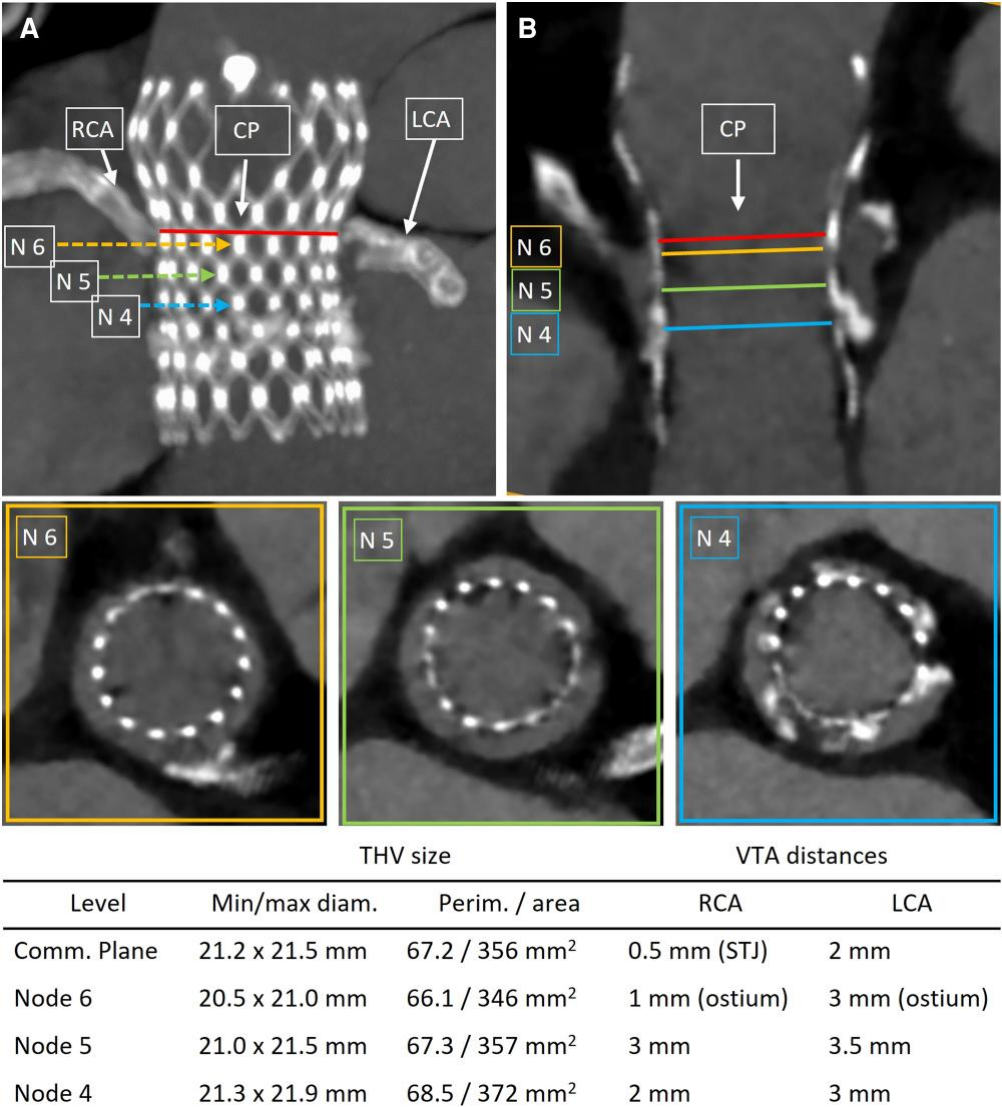
Step-by-step computed tomography analysis of index transcatheter aortic valve replacement. Identification of Evolut transcatheter heart valve commissural plane (red line), located above the coronary ostia, and identification of Evolut transcatheter heart valve node 6, 5, and 4 planes in a maximum intensity projection (*A*) and a longitudinal view (*B*). Axial view at Evolut transcatheter heart valve node 6 (N6), showing a prohibitive valve-to-aorta distance to right coronary artery. Axial views at Evolut node 5 (N5) and node 4 (N4), displaying more permissive valve-to-aorta distances. The table below summarizes Evolut transcatheter heart valve size and valve-to-aorta distances at each level. CP, commissural plane; CT, computed tomography; LCA, left coronary artery; RCA, right coronary artery; N6, node 6; N5, node 5; N4, node 4; VTA, valve-to-aorta; THV, transcatheter heart valve.

**Figure 2:**
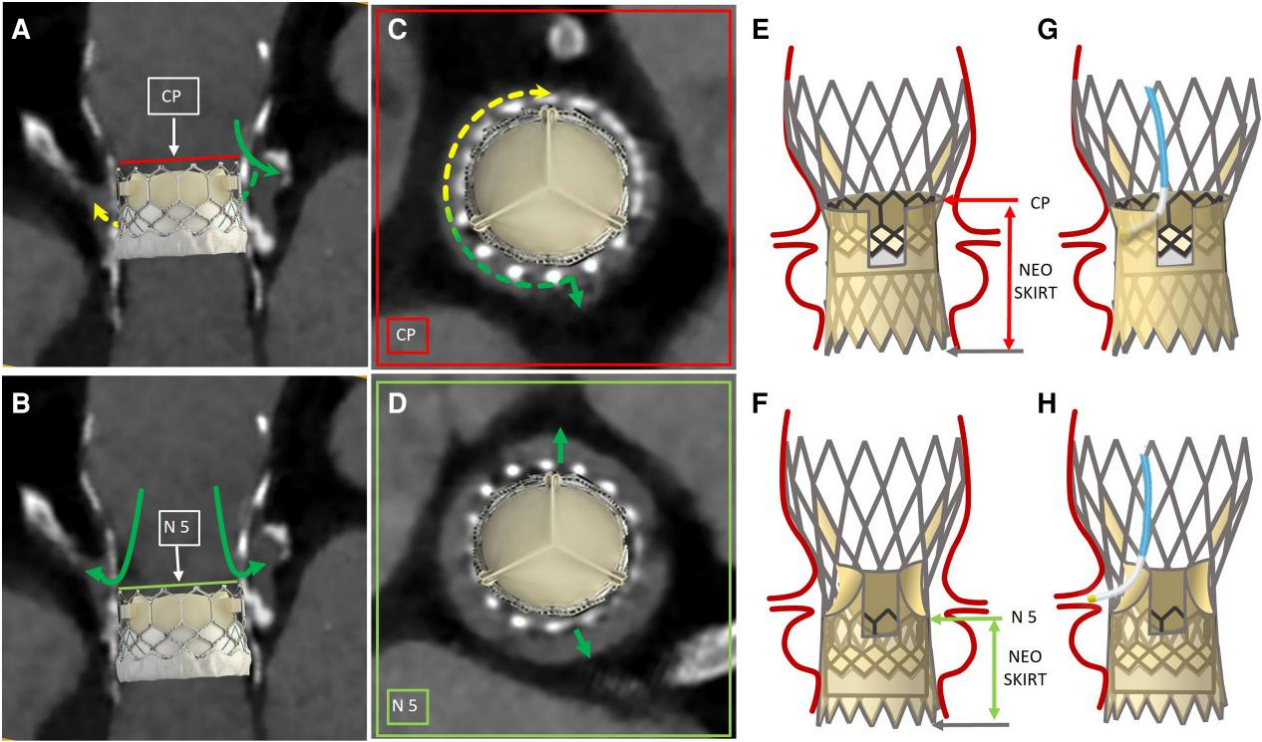
Pre-transcatheter aortic valve replacement-in-transcatheter aortic valve replacement planning. Reconstruction of intra-annular (*A*) and Evolut transcatheter heart valve node 5 (*B*) redo-transcatheter aortic valve replacement with SAPIEN 3 transcatheter heart valve. Commissural plane is drawn in red, green, and yellow arrows illustrate direct and indirect coronary artery perfusion after redo-transcatheter aortic valve replacement. Axial view at commissural plane (*C*) and at Evolut node 5 (*D*) illustrating the residual free distance available for coronary perfusion after redo-transcatheter aortic valve replacement. Note that with high SAPIEN transcatheter heart valve implantation, right coronary sinus would receive only indirect perfusion flow, from outside Evolut transcatheter heart valve frame. Schematic illustrations of the two different levels of implantation (*E* and *F*). In intra-annular redo-transcatheter aortic valve replacement, the neoskirt reaches the Evolut commissural plane (red arrow, *E*); in case of low SAPIEN transcatheter heart valve implantation, the smaller neoskirt allows Evolut leaflet overhang (*F*). Schematic illustrations of coronary access after intra-annular (*G*) and low implantation redo-transcatheter aortic valve replacement with Evolut leaflet overhang (*H*). CP, commissural plane; N5, node 5; THV, transcatheter heart valve.

An 80-year-old man, who 6 years earlier underwent a TAVR with a CoreValve Evolut R 29 mm (Medtronic Inc., Minneapolis, MN, USA), was admitted to our centre for acute heart failure. His coronary history included three coronary artery bypass grafts and a more recent PCI. Comorbidities included advanced chronic renal insufficiency (estimated glomerular filtration rate 24 mL/min/1.73 m^2^) and severe polyvasculopathy. Subsequent evaluation at our institution revealed severe intraprosthetic aortic insufficiency, suggestive of acute flail/rupture of the left neo-cusp and a severely reduced ejection fraction [left ventricular ejection fraction (LVEF) 32%] (*Video 1*). Coronary angiography confirmed a three-vessel coronary artery disease with patent left internal mammary artery graft to left anterior descending and patent stents on left main–left circumflex artery and right coronary artery (RCA) (Supplementary material online, *Video S1*). The CT scan for procedural planning revealed short VTA distances near the RCA (VTSTJ 0.5 mm, VTC 1 mm, *Figure 1*), while they were more permissive near the left coronary artery (VTSTJ 2 mm, VTC 3 mm, *Figure 1*). Although the implantation of the index Evolut THV resulted moderately deep, the commissural plane was located above both coronary ostia (*Figure 1*, between Evolut THV node 6 and node 7). Consequently, crushing the entire Evolut leaflets would have created a covered cylinder extending up to the coronary ostia, minimizing specifically RCA perfusion and rendering future CA impracticable (*Figure 2*). To reduce the neoskirt height, we selected a short-stent frame SAPIEN 3 THV (Edwards Lifesciences Inc., Irvine, California). We planned to implant it with alignment below the Evolut THV node 6 to obtain index leaflet overhang, guided by multiplanar CT (*Figures 1* and *2*) and real valve models reconstructions (Supplementary material online, *Videos S2* and *S3*). The sizing of the SAPIEN THV was tailored to the minimal internal diameter of the Evolut stent frame, measuring 20.5 mm at the level of Evolut THV node 6 and 21.3 mm at its node 4 (*Figure 1*). We decided to implant a 23 mm SAPIEN 3 prosthesis, slightly deflated (−2 mL) to avoid expansion of the failed index Evolut THV and thereby mitigating the risk of RCA obstruction. The patient’s clinical condition deteriorated, with pulmonary oedema occurrence and signs of low cardiac output. Urgent TAVR-in-TAVR procedure was performed under general anaesthesia and transoesophageal echocardiogram (TOE) guidance. After crossing of the aortic valve with a guidewire, the patient experienced severe hypotension requiring inotropic support. Due to haemodynamic instability, we did not use any coronary protection. A 23 mm SAPIEN 3 THV was implanted within the Evolut THV stent frame at node 5, below the coronary ostia, with immediate improvement of haemodynamic conditions. No changes in electrocardiogram and in ventricular wall motion were documented. TOE evaluation revealed no transvalvular gradient, no paravalvular leak (PVL) or intravalvular regurgitation (*Video 2*). Aortic angiography confirmed the presence of proper coronary perfusion (*Video 3*). The patient’s haemodynamic status stabilized; he was extubated on the same day of the procedure. The patient was discharged in good clinical status (NYHA II). Pre-discharge echocardiography demonstrated the absence of aortic regurgitation with normal trans-prosthetic gradient (mean gradient 9 mmHg) and reduced ejection fraction (LVEF 35%). The 6-month follow-up visit confirmed the proper performance of the aortic THV (mean gradient 9 mmHg) without PVLs, with an improvement in LVEF (42%) and systolic pulmonary arterial pressure (35 mmHg).

## Discussion

Planning TAVR-in-TAVR procedures builds on the recognition of anatomical interactions between the aortic root, coronary ostia, and the degenerated THV.^1–4^ With supra-annular THVs, the commissural plane is higher; therefore, the VTA distances and STJ dimensions become crucial to prevent coronary occlusion and sinus sequestration. According to the previous literature, a redo-TAVR with index THV commissural plane placed above the coronary ostia and a VTA < 2 mm is burdened by prohibitive risk of coronary artery obstruction.^2,6,7^ Recently, a lower implantation of a short-stent frame THV inside a first tall-stent frame supra-annular THV has been suggested, allowing the index THV leaflet overhang and therefore reducing the neoskirt height.^4,5^ Specific to the treatment of an Evolut R THV, redo-TAVR with a low SAPIEN 3 THV implantation has been tested *in vitro* with different degrees of leaflet overhang and no significant compromise to valve function.^5^ The current case represents, to the best of our knowledge, the first-in-human application of redo-TAVR with low SAPIEN 3 THV implantation within an Evolut R THV obtaining index leaflet overhang, preserving coronary perfusion and potential coronary re-engagement. Additionally, we confirmed *in vivo* that the unjailed superior half of the Evolut leaflets did not haemodynamically interfere with the normal function of the second THV (see Supplementary material online, *Figure S1*). We undersized the SAPIEN 3 THV compared to the study *in vitro* (23 mm S3 instead of 26 mm S3,^5^ within a 29 mm Evolut R THV) adapting to the index Evolut THV diameters observed to the CT scan (20.5 mm waist instead of theoretical 23 mm waist). The expansion of the index Evolut frame with a 26 mm SAPIEN THV may have limited space in the sinus of Valsalva, increasing the risk of sinus sequestration and right coronary obstruction (1.8 mm expansion for a 26 mm SAPIEN inside a 29 mm Evolut R THV measured *in vitro*.^5^)

Thanks to deep index Evolut THV implantation, the right coronary ostium (the lower of the two) was located at the level of node 6 (*Figure 1*). Thereby, we planned to implant the SAPIEN THV at node 5, limiting leaflet overhang to what was essential to prevent coronary occlusion.

Theoretically, reducing the neoskirt height and allowing for a greater extent of leaflet overhang could enhance coronary perfusion and access through the frame of the Evolut THV. While implantation of the SAPIEN THV at the Evolut THV’s node 4 has been explored *in vitro* with intact THVs,^5^ a more pronounced overhang from degenerated and calcified leaflets might elevate the risk of dysfunction of the secondary THV. Finally, long-term clinical implications and long-term performance data of leaflet overhang are missing.

## Conclusions

Following *in vitro* study, this case demonstrates *in vivo* feasibility of redo-TAVR with low SAPIEN 3 THV implantation within an Evolut R THV obtaining index leaflet overhang. Moreover, index leaflet overhang proved to preserve coronary perfusion and the second THV hydrodynamic performance.

## Lead author biography


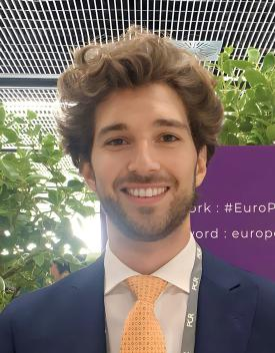
Dr Tommaso De Ferrari, MD, is an interventional cardiologist fellow at San Raffaele University Hospital of Milan, Italy (Director Prof. M. Montorfano). He trained as a cardiology fellow at the University of Messina, Italy. His areas of interest include coronary and structural interventions.

## Supplementary Material

ytae529_Supplementary_Data

## Data Availability

All data generated or analysed during this study are included in this published article and its supplementary information files.

## Supplementary material

**Consent:** The authors confirm that the EHJ-CR Consent Form has been signed by the patient and that the original of the signed form is held by the treating institution.

## Notes

**Funding:** None declared.
