# Supplementary material for: Transcatheter aortic valve replacement-in-transcatheter aortic valve replacement for high-risk anatomies: demonstrating the feasibility of index leaflet overhang in a first-in-human case report
Source: Eur Heart J Case Rep. 2024 Sep 24;8(10):ytae529. doi: 10.1093/ehjcr/ytae529 (PMC11635632; doi:10.1093/ehjcr/ytae529)
Supplement: ytae529_Supplementary_Data [file ytae529_Supplementary_Data.zip › Supplemental Appendix.docx]

**Supplementary Material**

**Supplementary Figure 1: illustration of low SAPIEN THV implantation and Evolut leaflet overhang. (A)** Schematic figure illustrating the risk of interference during diastole between the unjailed superior half of the Evolut leaflets and the SAPIEN leaflets below. Green arrows represent blood flow pushing the Evolut leaflets over the SAPIEN leaflets, thereby creating aortic regurgitation (red arrows). **(B)** Ideal performance of Evolut leaflet overhang: blood flow (green arrows) pushes down the SAPIEN leaflets ensuring the normal closure of the transcatheter heart valve during diastole.

**Supplementary Video 1: Coronary angiography.** It confirmed three-vessel coronary artery disease with patent stents on left main coronary artery (LM), left circumflex coronary artery (LCx) and right coronary artery (RCA). Coronary access occurs through the Evolut stent frame, below the Commissural Plane.

**Supplementary Video 2 and 3: Real valve models reconstructions.** Reconstruction of Intra-annular (Supplementary Video 2) and low implantation with index leaflet overhang (Supplementary Video 3) Redo-TAVR with 23-mm SAPIEN 3 and 29-mm Evolut R THV models, testing subsequent coronary access.
