# Supplementary figures and images for: Transcatheter aortic valve replacement-in-transcatheter aortic valve replacement for high-risk anatomies: demonstrating the feasibility of index leaflet overhang in a first-in-human case report
Source: Eur Heart J Case Rep. 2024 Sep 24;8(10):ytae529. doi: 10.1093/ehjcr/ytae529 (PMC11635632; doi:10.1093/ehjcr/ytae529)

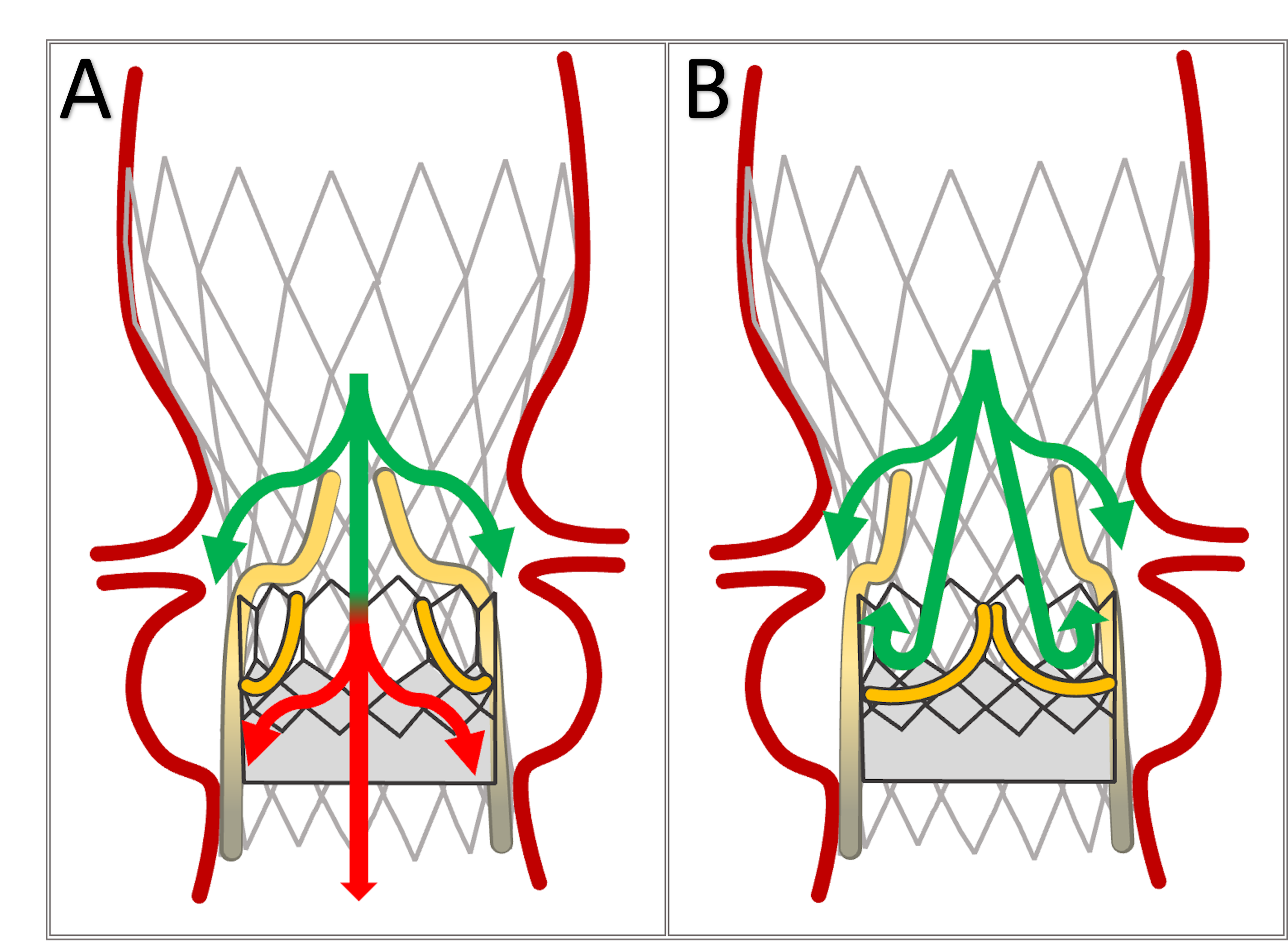

Supplement: ytae529_Supplementary_Data [file ytae529_Supplementary_Data.zip › Supplementary_Figure_1.tif]
